# Adaptation to feedback representation of illusory orientation produced from flash grab effect

**DOI:** 10.1038/s41467-020-17786-1

**Published:** 2020-08-06

**Authors:** Yijun Ge, Hao Zhou, Chencan Qian, Peng Zhang, Lan Wang, Sheng He

**Affiliations:** 1 State Key Laboratory of Brain and Cognitive Science, Institute of Biophysics, Chinese Academy of Sciences, 100101 Beijing, China; 2 Department of Psychology, University of Minnesota, Minneapolis, MN 55455 USA; 3 University of Chinese Academy of Sciences, 100049 Beijing, China; 4 Sino-Danish Center for Education and Research, 100190 Beijing, China; 5 CAS Center for Excellence in Brain Science and Intelligence Technology, 20003 Shanghai, China

**Keywords:** Neuroscience, Psychology

## Abstract

Adaptation is a ubiquitous property of sensory systems. It is typically considered that neurons adapt to dominant energy in the ambient environment to function optimally. However, perceptual representation of the stimulus, often modulated by feedback signals, sometimes do not correspond to the input state of the stimulus, which tends to be more linked with feedforward signals. Here we investigated the relative contributions to cortical adaptation from feedforward and feedback signals, taking advantage of a visual illusion, the Flash-Grab Effect, to disassociate the feedforward and feedback representation of an adaptor. Results reveal that orientation adaptation is exclusively dependent on the perceived rather than the retinal orientation of the adaptor. Combined fMRI and EEG measurements demonstrate that the perceived orientation of the Flash-Grab Effect is indeed supported by feedback signals in the cortex. These findings highlight the important contribution of feedback signals for cortical neurons to recalibrate their sensitivity.

## Abstract (web summary)

Feedforward-feedback signal interactions are common in the brain during sensory information processing. Here, the authors show that feedback-driven representation of perceived orientation dominates visual adaptation, despite the discrepant feedforward representation of input orientation.

## Introduction

Though adaptation is typically considered to be neurons adjusting their sensitivity to accommodate to the state of the “world”^1,2^, it is necessarily the case that the state of the “world” is reflected in neural representations. However, neural processing involves both feedforward as well as feedback signals, typically with the feedforward signal more directly representing the proximal stimulus^3^ while the feedback signal, influenced by spatiotemporal contextual factors, leading to the perceptual representation of the distal stimulus. In sensory information processing, contextual modulation and feedforward–feedback interactions are very common^4–6^. An important unresolved question is whether the feedforward or feedback-driven representation determines the outcome of cortical neuronal adaptation, especially when they are discrepant.

To address this question, it is necessary to dissociate the input feedforward signals from cortical feedback signals in the brain. A recently discovered visual illusion, flash-grab effect (FGE)^7^, provides such an opportunity. The FGE occurs when a bar is briefly flashed on the light–dark boundary of a sectored background moving back and forth, at the time-point of direction reversal of background motion. The “flashed” bar could be perceived as tilted by more than 10° away from its original orientation, as what would be perceived without the moving background inducer^7^.

Since the FGE can alter perceived orientation, an orientation-specific adaptation was adopted to investigate whether adaptation would be based on the original retinal or perceived orientation. The tilt-aftereffect (TAE) is a robust visual phenomenon that results from orientation-selective adaptation of visual neurons^8^. After prolonged exposure to an adaptor slightly tilted from vertical, a vertical test is perceived as tilted away from the adapting orientation^9^. The underlying mechanism of this aftereffect was thought to be that cortical orientation-selective neurons in the visual system adjust or recalibrate their sensitivity based on the prevalent orientation and contrast of incoming signals, often in a population coding context, and with the goal of achieving more efficient coding^2,8,10–18^.

Testing of the TAE with the FGE will inform us about the relative contribution to orientation adaptation from the input retinal orientation and the contextual modulated perceived orientation. However, for our goal, we would also need to establish a close link between the perceived orientation of FGE and the feedback signals. While previous neuroimaging experiments showed that the perceived orientation in the FGE could be decoded in the retinotopic cortex^19^, it remains unclear how the neural signals dynamically support the perceived orientation of the flashed bar^20^. Thus, we performed high spatial and temporal resolution human brain imaging experiments to delineate the dynamic contribution of feedforward and feedback signals to the perceived orientation in FGE. As shown in the results section, we obtained strong evidence that the perceived orientation in FGE was indeed supported by feedback signals. With this link established, a demonstration of TAE from the perceived orientation would indicate that the feedback signals dominate cortical adaptation.

In the following sections, we first present behavioral data showing that perceived orientation dominates the TAE. Then, we show results from high spatial-temporal resolution measurements of the cortical representation of the perceived orientation in FGE. The time-resolved EEG data and layer-resolved fMRI data provide clear evidence that the perceived tilt in FGE is driven by late onset feedback signals, primarily targeting the superficial layers of the retinotopic cortex. These results together strongly suggest that perceived orientation in FGE is supported by feedback signals in the early visual cortex, which dominate orientation-selective adaptation in spite of the available feedforward signal corresponding to the original orientation of the flashed bar stimulus on the retina.

## Results

### TAE depends on the perceived rather than retinal input orientation

In two psychophysics experiments, we investigated the relative contribution of the perceived vs. retinal orientation of FGE to the TAE. In the first experiment, the adapting bars vertical at the retinal level were perceived as tilted away from vertical orientation; in the second experiment, the adapting bars tilted at the retinal level were perceived as vertical. In both experiments, the testing bars were presented around the vertical orientation.

**Fig. 1:**
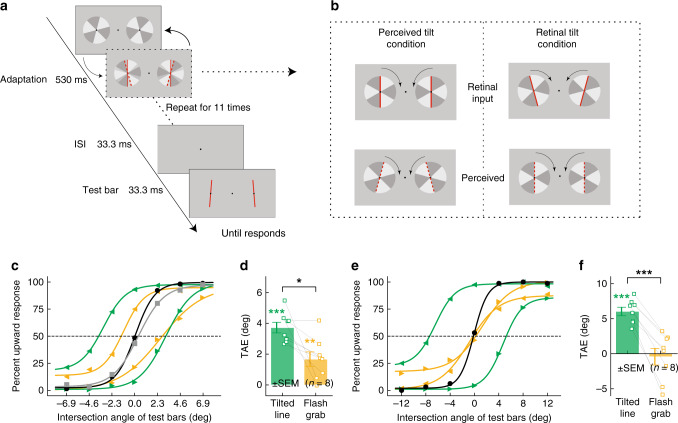
Psychophysics stimuli and results. **a** Stimulus presentation sequence for the TAE measurement. **b** The stimuli used in the two orientation adaptation conditions and demonstrations of subjects’ illusory perception. Left part: original vertical but perceived tilt with flash grab inducer (tilt angle is by 15.55° on average (SD = 7.54)). Right part: original tilt but perceived vertical with flash grab inducer. **c** Fitted psychometric functions using logistic regression in experiment 1, **d** Averaged TAE sizes across subjects (*n* = 8) for conventional tilted bars condition (*p* < 0.001 Holm-corrected) and flash grab condition (*p* = 0.010 Holm corrected) in experiment 1 (two-sided paired t-test, *p* = 0.013, *d* = 1.17). **e** Fitted psychometric functions using logistic regression in experiment 2. **f** Averaged TAE sizes across subjects (*n* = 8) for conventional tilted bars condition (*p* < 0.001 Holm corrected) and flash grab condition (*p* = 0.730 Holm corrected) in experiment 2 (two-sided paired t-test, *p* < 0.001, *d* = 2.80). Black curve: vertical bars only condition; gray curve: rotating background-only condition; green curves/bars: flashed tilted bars only condition (conventional tilt adaptation); orange curves/bars: flash grab illusion condition. Error bars indicate standard errors of the mean (*n* = 8 (individual subject)). Source data are provided as a Source Data file.

Subjects viewed a pair of vertical bars that were repeatedly and briefly flashed on top of two patterned disks that oscillated clockwise and counter-clockwise, with the flashed bars presented at the moment of the rotation reversals. The adapting bars, which would be perceived as vertical if presented without the moving background inducer, were perceived as tilted away from vertical due to the FGE (Fig. 1a, and left column of 1b). On each trial of the main experiment condition, subjects were presented with 11 flashes (10.6 s) of adaptation, followed by 33.3 ms of blank screen, then the test bars for 33.3 ms (Fig. 1a). Subjects were asked to judge whether two test bars converged upward or downward using a two-alternative forced choice (2AFC) method. Three control adaptation conditions were also included in the experiment: (a) the vertical flashed bars only, without the rotating background disks; (b) the rotating background disks only; (c) tilted (5.71° from vertical) flashed bars only, without the rotating background disks. The four conditions were presented in separate blocks.

Results show that a significant TAE was generated by perceptually tilted bars: both the tilted bars without moving background and the FGE-induced tilted bars. Figure 1c shows the psychometric functions for adaptation to the FGE and the other three control conditions. Not surprisingly, there was no TAE in both the no background, vertical bar only condition (*p* = 0.956) and the background-only condition (*p* = 0.534). The strength of the TAE could be measured as half the difference on the *x*-axis between the two points of subjective equality (PSEs) following adaptation in two opposite orientations, i.e., the distance between the two green or two orange fitted curves (Eq. (1)). Figure 1d plots the magnitude of the TAE from the flashed tilted bar adaptors (conventional TAE) and the TAE from the FGE condition. As expected, the conventional tilt adaptation condition generated strong TAE (*M* = 3.72 deg, SD = 0.97, *t*(7) = 10.80, Holm-corrected *p* < 0.001, *d* = 3.84). The key result here is that a significant TAE was observed in the flash grab condition (*M* = 1.67 deg, SD = 1.34, *t*(7) = 3.53, *p* = 0.010 corrected, *d* = 1.25), though it was weaker than the conventional TAE (two-sided paired sample t-test, *t*(7) = 3.31, *p* = 0.013, *d* = 1.17).

The first experiment demonstrates that perceived tilted orientation could induce a TAE even though the input retinal orientation was vertical. Does the input orientation contribute to the TAE separately from the perceived orientation? To address this question, we tested subjects who adapted to bars with tilted input orientation but were perceptually vertical due to FGE (Fig. 1b, right panel). At the beginning of this experiment, each individual subject adjusted the orientation of the flashed bars in FGE condition so that the bars were perceived as vertical. The adjusted retinal orientation was then set as the input orientation of adapting condition under FGE. Similar to the first experiment, we also included two control conditions: the vertical bars only condition and the tilted bars only condition.

Figure 1e, f shows the results of this experiment. The two control conditions generated results as expected: without the moving background inducer, the vertical bars by themselves did not generate the TAE (*p* = 0.367), and the tilted bars generated very robust TAE (*M* = 6.02 deg, SD = 1.69, *t*(7) = 10.08, *p* < 0.001 corrected, *d* = 3.56). However, with the moving background inducer, the key result is that the originally tilted but perceptually vertical bars (due to FGE) generated no measurable TAE (*M* = −0.43 deg, SD = 3.36, *t*(7) = −0.36, *p* = 0.730 corrected), which is significantly weaker than the conventional TAE (two-sided paired sample t-test, *t*(7) = 7.91, *p* < 0.001, *d* = 2.80) as shown in Fig. 1f.

Results from the two psychophysics experiments clearly show that, when the adaptor’s perceived orientation is dissociated from its input orientation, the TAE is induced by the perceived rather than the input orientation itself. In other words, orientation-selective adaptation seems to be primarily based on the eventual perceptual representation of the stimuli rather than simply on the neural representation directly linked to the input signals. To further understand the contribution of feedforward and feedback signals to FGE and in turn to orientation-selective adaptation, we conducted fMRI and ERP studies investigating the spatial and temporal neural correlates of the FGE.

### Representation of FGE in the retinotopic visual cortex

We investigated the neural representation of the FGE in retinotopic visual areas in two fMRI experiments. The first experiment was conducted on a 3T scanner, with a focus on the retinotopic representation of the flashed bar under FGE. The second experiment was performed at high spatial resolution on a 7T scanner, which allowed us to obtain layer-resolved response signals to FGE in the retinotopic visual cortex. With known biases of feedforward and feedback signals in different cortical layers, the 7T data could inform us about the relationship between feedback signals and perceptual representation.

**Fig. 2:**
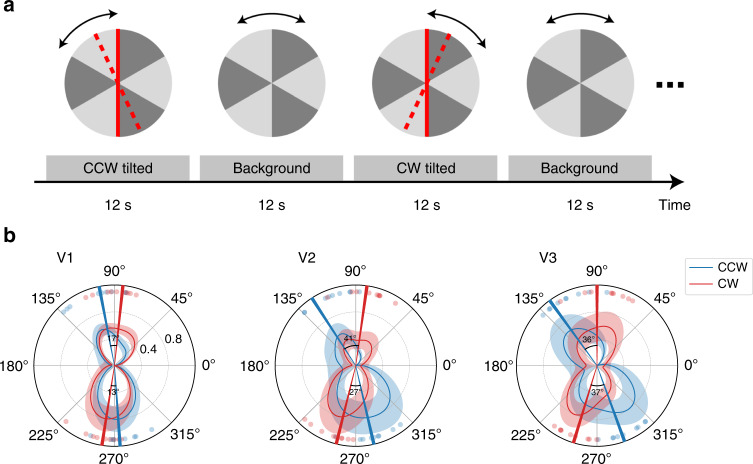
Stimuli and results of the 3T fMRI. **a** Schematic diagram of stimuli and procedures for the 3T fMRI experiment. A red bar flashed repeatedly for 12 s at the reversal point of the background motion, alternating with 12 s background-only stimulation. The bar was presented at the vertical meridian but would be perceived as tilted clockwise or counter-clockwise from the vertical, depended on the direction of motion reversal. Red solid lines indicate the original position of the bar, while red dotted lines illustrate subjects’ perception of the bar. **b** Polar angle representation of the flashed bar in FGE in the early visual cortex. Normalized fMRI response to the clockwise and counter-clockwise tilted illusions were plotted as a function of polar angle coordinates across the visual field. The minimal and maximum polar response for each subject were normalized to 0 and 1 (first subtracted the min and then divided by the max). Red and blue curves show the average polar response across subjects (low pass filtered by convolving with a 60°-width hamming window for illustration purposes). Shaded areas indicate standard errors of the mean (*n* = 9 (individual subject)). Red and blue bars illustrate the estimated average tilt from the curves, whereas the dots indicate the estimated tilt for individual subjects. Source data are provided as a Source Data file.

In the 3T fMRI experiment, we obtained the BOLD signal activated by the flashed bar in the FGE with block-designed fMRI scans (Fig. 2a shows the stimuli and procedure of the experiment). Subjects’ retinotopic maps were also obtained using the standard rotating wedge and expanding/contracting ring stimuli^21^ in two separate scans. The retinotopic map provides, for each voxel in the early visual cortex, the polar angle coordinate of its population receptive field. fMRI responses to the flashed bar for voxels with the same polar angle preference were averaged and used as the radial coordinate, plotted as a function of polar angle across the visual field (Fig. 2b). From V1 to V3, the fMRI response to the clockwise FGE was stronger in the upper right and lower left quadrants of the visual field in comparison with the counter-clockwise illusion, which showed stronger responses in the upper left and lower right quadrants. Therefore, the retinotopic representation of FGE in the early visual cortex is qualitatively consistent with the perceived tilt of the flashed bar. We further estimated the angular difference between the two polar angle representations of fMRI signals in the visual cortex. Note that the angular difference represents the summed effect of clockwise and counter-clockwise tilts. The estimated angular difference was smaller in V1 (17° and 13° for upper and lower visual field, respectively) compared to V2 (41 and 27°) and V3 (36° and 37°) (Fig. 2b). One-way ANOVA showed that the illusory effect significantly varied across visual cortical areas (*F*(2, 16) = 22.24, *p* < 0.001, $\eta _p^2 = 0.735$). Post hoc analysis showed that the illusory effect was significantly stronger in extra-striate than in striate visual cortex (for V2, *t*(8) = 5.47, *p* < 0.002, *d* = 1.824; for V3, *t*(8) = 5.50, *p* = 0.002, *d* = 1.834), while no significant difference was observed between V2 and V3 (*t*(8) = 2.07, *p* = 0.072). An important consideration is that BOLD responses reflected both the feedforward and feedback influences, and the reason for the smaller estimated tilt representation in V1 could be that V1 activity had a greater contribution from feedforward input signals (corresponding to the retinal orientation). The relative contribution of feedforward vs. feedback signals in different areas was investigated further with layer-resolved imaging^22–25^ as described in the following 7T high-resolution fMRI experiment.

In the follow-up 7T fMRI experiment, we obtained high-resolution layer-specific representation of the FGE in different layers of V1 to V3. The paradigm was essentially the same as the 3T experiment, with the exception that the flashed bar was presented on the horizontal rather than vertical meridian due to the limited vertical field of view imposed by the 7T coil (Supplementary Fig. 2). In three independent scans, subjects were presented with a rotating bar (centered on the fixation point) to map the polar angle retinotopy of early visual areas^21^. In the following layer-resolved analysis, the original fMRI data were resampled from 0.85 or 0.8 mm to 0.4 mm isotropic voxel size. Voxels were separated based on their distances from cortical surfaces into three separate layers: from 0% to 40% the superficial layers (S), from 40% to 80% the middle layers (M), and from 80% to 100% the deep layers (D)^22,23,26^. Responses in each ROI to the clockwise and counter-clockwise tilted illusory orientations under FGE were plotted for voxels tuned to different orientations. For each layer (S, M, or D), there were two response curves, one corresponding to the perceived clockwise tilted and the other to the counter-clockwise tilted bars (Supplementary Fig. 3). To alleviate the bias of BOLD response towards superficial layers, the response curves were normalized across conditions within each cortical layer.

**Fig. 3:**
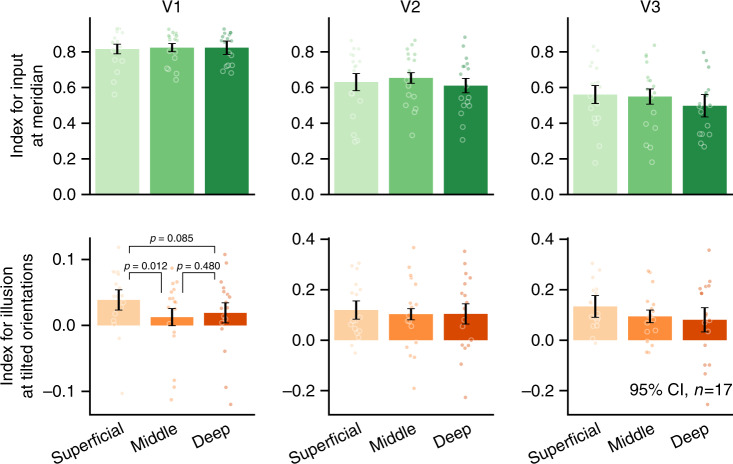
Representations corresponding to retinal input and illusory percepts across different layers of early visual cortices. fMRI response to the clockwise and counter-clockwise tilted illusions were calculated as a function of bar angle coordinates across the field of bar rotation. The computed response index for input representation at the horizontal meridian was based on the mean BOLD responses between −4° to 4° polar angles, while the illusory index was based on the mean BOLD response differences between two experimental conditions (clockwise vs. counter-clockwise) at both −14° to −6° and 6° to 14° polar angles. The representation index for the “illusory orientation” was significantly stronger in V2 and V3 than in V1 (two-way repeated measure ANOVA, *F*(2, 32) = 9.72, *p* < 0.001; Post hoc Holm corrected, V1 vs. V2: *t*(16) = −3.23, *p* = 0.010, *d* = −0.783, V1 vs. V3: *t*(16) = −4.14, *p* = 0.002, *d* = −1.005, V2 vs. V3: *t*(16) = 0.31, *p* = 0.755), consistent with the 3T fMRI results; in contrast, the strength of signal corresponding to the input orientation (horizontal) was much stronger in V1 than in V2 and V3 (two-way repeated measure ANOVA, *F*(2, 32) = 27.76, *p* < 0.001; Holm corrected post hoc, V1 vs. V2: *t*(16) = 4.84, *p* < 0.001, *d* = 1.176, V1 vs. V3: *t*(16) = −6.94, *p* < 0.001, *d* = 1.685, V2 vs. V3: *t*(16) = 2.61, *p* = 0.019, *d* = 0.634). When signals were analyzed from different layers, the illusory representation varied significantly across layers in V1 (*F*(2, 32) = 3.91, *p* = 0.030). Significant illusory representation was observed in V1 superficial layer (*t*(16) = 3.16, *p* = 0.018 Bonferroni corrected), bu*t* not in V1 middle layer (*t*(16) = 0.88, *p* = 0.392), and post hoc comparison showed that the illusory effect was significantly stronger in V1 superficial layer than in the middle layer (*t*(16) = 2.81, *p* = 0.037 Holm corrected). The sta*t*istical com*p*arison across layers in V1 (one-way repeated measure ANOVA) was conducted by “within-subject” design. All the error bars represent within-subject 95% confidence interval of the mean index (*n* = 17 (individual subject)). Source data are provided as a Source Data file.

We calculated indexes that reflect the signal strength corresponding to the input meridian orientation and perceived tilted orientation respectively, for different layers and separately for V1, V2, and V3 based on the normalized response curves. Specifically, the index for the perceived orientation was calculated based on the mean BOLD response differences between two experimental conditions (clockwise vs. counter-clockwise) over the range of −14° to −6° and 6° to 14° polar angles. The index for input meridian orientation signal was calculated based on the mean BOLD response between −4° and 4°. As shown in Fig. 3, the main effects are: 1) The representation index for the “illusory orientation” was significantly stronger in V2 and V3 than in V1 (*F*(2, 32) = 9.72, *p* < 0.001, $\eta _p^2 = 0.378$); in contrast, the strength of signal corresponding to the input horizontal orientation was much more robust in V1 than in V2 and V3 (*F*(2, 32) = 27.76, *p* < 0.001, $\eta _p^2 = 0.634$). 2) More importantly, when signals were analyzed from different layers, the illusory representation varied significantly across layers in V1 (*F*(2, 32) = 3.91, *p* = 0.030, $\eta _p^2 = 0.196$). Significant illusory representation was observed in V1 superficial layer (*t*(16) = 3.16, *p* = 0.018 Bonferroni corrected, Cohen’s *d* = 0.766), but not in V1 middle layer (*t*(16) = 0.88, *p* = 0.392), and post hoc comparison showed that the illusory effect was significantly stronger in the superficial layer than in the middle layer (*t*(16) = 2.81, *p* = 0.037 Holm corrected, Cohen’s *d* = 0.682). These layer-specific results indicate that the neural representation of FGE is primarily localized in the superficial layer for V1, but not the middle layer. This is consistent with previous studies showing that responses in the V1 middle layer reflect mainly bottom-up input signals, while responses in the V1 superficial layers are more related to feedback signals^27–31^. In other words, the layer-resolved 7T data of FGE suggest that the representation of the perceived tilt was likely driven by feedback signals.

### FGE correlates with late visual evoked potential signals

While the fMRI results suggest that early visual areas are closely involved in FGE representation, with the 7T layer-resolved data suggesting a dominant feedback contribution to the FGE, the temporal dynamics of feedforward and feedback processing in FGE remain unclear. Thus, we adopted EEG measurements to address this question.

**Fig. 4:**
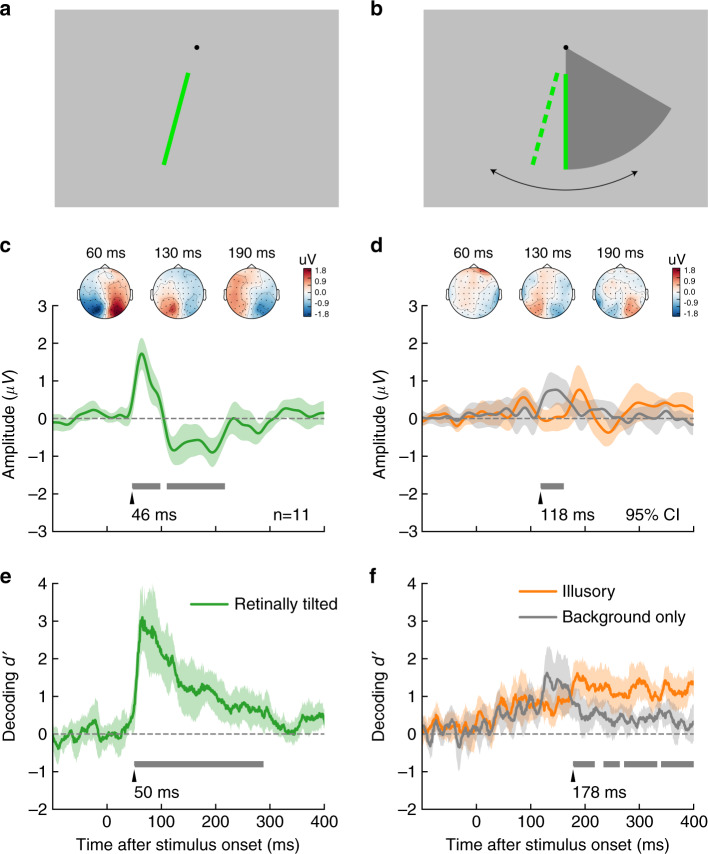
Stimuli and results of the EEG experiment. **a**, **b** Visual stimulus and perception (dashed bar) in tilted bar only (**a**) and illusory (**b**) conditions. **c**, **d** The differential ERPs (contralateral minus ipsilateral) averaged over five posterior electrodes and all subjects (n = 12 (individual subject)) in the tilted bar only (green), illusory (orange), and background-only (gray) conditions. Insets show corresponding topography snapshots in three diagnostic time points, assuming the bar flashed to the left of the vertical meridian. Note the topography in (**d**) corresponds to differential wave between illusory and background-only conditions. **e**, **f** Cross-validation performance of linear classifiers trained at each time point to predict to which side the bar was flashed (green), was perceived (orange), or would have been perceived if flashed (gray) averaged across subjects. Gray bars indicate the significant time period after multiple comparison correction (*p* threshold = 0.05) Error bands indicate 95% confidence interval obtained by bootstrap. Source data are provided as a Source Data file.

Considering the limited spatial resolution of EEG, the flashed bar was only presented in the lower visual field so that perceptually with the influence of FGE, the flashed bar would fall onto either the left or right visual field (Fig. 4b). This meant that an invoked ERP signal corresponding to the perceptual representation would be lateralized. In essence, the timing of the lateralized component in the ERP signal should indicate the timing of the neural representation of the perceptual effect. Trials with only a rotating background were included as a baseline condition, and trials with only a retinally tilted flashed bar without the rotating background were also included as a control condition. The orientation of the retinally tilted flashed bar were individually adjusted to roughly match the perceived orientation in the FGE condition (Fig. 4a).

Figure 4c, d show the differential ERP from posterior electrodes evoked by the contralateral versus ipsilateral bar in all three conditions. As expected, we observed a clear lateralized C1 component in retinally tilted condition (Fig. 4c), in response to the lateralized feedforward input. The cluster-based permutation test revealed an early positive peak (46–98 ms) within C1 latency and a later negative peak (110–217 ms). In contrast, after subtracting the background-only condition, no corresponding lateralized C1 was found in the illusory condition (Fig. 4d), but only the later negative peak (118–161 ms) remained, at which time window the rotating background generated a positive deflection. This is consistent with the lack of lateralized representation in the early visual cortex during the feedforward sweep.

**Fig. 5:**
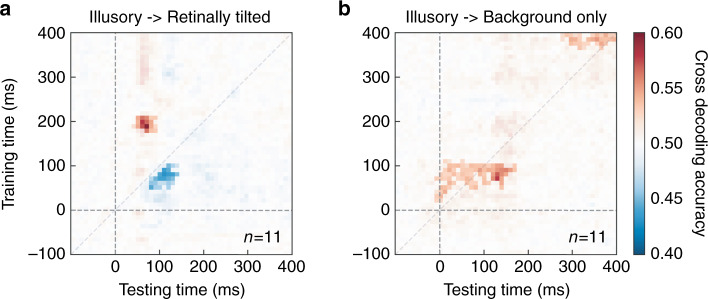
Cross-decoding analysis results. The color in each cell of the matrix indicates decoding accuracy if the classifier was trained with data from one time in one condition, and tested with data from another time in another condition. **a** Trained with illusory and tested with retinally tilted condition. **b** Trained with illusory and tested with background. The highlighted cells were significantly different (*p* threshold = 0.05) from chance level (50%) according to cluster-based permutation test, corrected for multiple comparisons. Source data are provided as a Source Data file.

We then performed multivariate pattern analysis to uncover the dynamic change of lateralized representation for the retinally or illusorily tilted stimuli from beyond the posterior electrodes. Linear classifiers were trained to predict whether the flashed bar was perceived to be tilted left or right at each time point (and for background-only trials, we were effectively predicting rotation direction). Retinally tilted trials could be decoded significantly above chance about 50 ms after stimulus onset, reaching peak performance at C1 latency (Fig. 4e). Illusory trials could also be successfully decoded starting from about 70 ms after stimulus onset. However, it outperformed the baseline condition (rotating background alone) only at a later stage, about 178 ms after stimulus onset (Fig. 4f). We further characterized the nature of the lateralization information in the illusory condition using the cross-decoding method. If the early lateralized representation before 100 ms reflected a mislocalized bar, similar to a retinally tilted one, then a classifier trained using data from illusory condition in this time period should be able to decode data from retinally tilted condition during C1 latency. The observed results did not support this hypothesis. Classifiers trained using illusory trials between 50–100 ms could not predict retinally tilted trials in the same period (Fig. 5a, the decoding accuracy was actually significantly below-chance level), but they did predict background-only trials significantly above chance level from 0 to around 150 ms (Fig. 5b). Importantly, stimulus side in retinally tilted trials between 50 and 100 ms could be predicted by classifiers trained using illusory trials between 180 and 220 ms (Fig. 5a). This suggests that the lateralized representation for the illusory tilt appeared at a relatively late stage, and the early information about stimulus side was more closely associated with the rotating background.

**Fig. 6:**
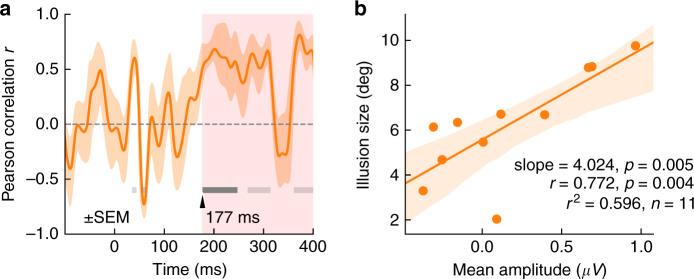
Inter-subject correlation analysis. **a** The Pearson correlation between instantaneous amplitude of the difference wave (contralateral minus ipsilateral) in illusory condition (with background-only condition subtracted) and illusion size was calculated at each time point. Dark gray bars indicate period with significant correlation (*p* < 0.05, cluster-based permutation test). Light gray bars indicate period when the absolute value of correlation was greater than the cluster-defining threshold *r* = 0.5. **b** The size of perceptual illusion (6.25 ± 2.35 deg) was well predicted by the mean amplitude, averaged within the shaded interval in (**a**). Source data are provided as a Source Data file.

We further asked whether and when lateralized EEG signals could predict the magnitude of the tilt perception in FGE. The inter-subject Pearson correlation between instantaneous amplitude of the difference wave (contralateral minus ipsilateral) in illusory condition (with background-only condition subtracted) and illusion size was calculated at each time point. Significant positive correlation emerged about 177 ms after stimulus onset (Fig. 6a). Figure 6b, based on the same data as the shaded region in Fig. 6a, more explicitly shows the clear relationship between the size of the perceptual illusion and the mean amplitude of the differential wave (*r*(9) = 0.77, *p* = 0.004). Notably, the onset of significant correlation matched well with the time when decoding performance in illusory condition overtook background-only condition, as well as when the scalp topography pattern in illusory condition became similar to that of retinally tilted condition in C1 latency, convergently supporting that the main relevant component for the illusory effect appears rather late, consistent with the typical timing of feedback signals.

In contrast to a robust and clearly lateralized C1 signal from the retinally tilted condition, no such lateralized signal was observed in the typical time window of C1 from the illusorily tilted bars under FGE. Only at a relatively late stage did the lateralized signal become prominent in the illusory condition, with its amplitude strongly correlated with the illusory effect size across individual subjects. These results support that the perceived tilt in FGE emerged later, likely a result of feedback processing.

Taken together, the fMRI results show that the distribution of fMRI BOLD signals in retinotopic visual cortical areas represented both the perceived and the input positions of the flashed bars. The 7T fMRI data further reveal that signals in the superficial layers were more influenced by the perceived illusory location of the flashed bars, especially in V1. Finally, a robust and behaviorally relevant lateralized EEG signature was only observed late in time, at around 170–180 ms after the onset of the flashed bars in the illusory condition. The combined spatiotemporal imaging results strongly suggest that the perceived tilt of the flashed bars in FGE was instigated by feedback signals.

## Discussion

The combined psychophysics, fMRI, and EEG results jointly support that cortical adaptation can be tuned to feedback-driven representations. In the case of orientation-selective adaptation investigated here, the TAE was mainly dependent on the perceived illusory orientation from the FGE rather than the input orientation of the flashed bar. With spatiotemporal imaging results supporting a feedback origin of the perceived orientation in FGE, these results suggest that feedback signals play an important role in orientation adaptation and provide evidence that in the presence of discrepant feedforward and feedback supported representation of visual input, the feedback signal determines the adaptation outcome.

A recent fMRI decoding study showed that patterns of activation in early visual cortex could be used to classify the direction of perceived position shift of FGE^19^. Our study went beyond decoding and (1) generated direct estimates of the angular representations of FGE in early visual cortex (3T fMRI), (2) identified the relative contributions from different cortical layers to the perceptual illusion (7T fMRI), and (3) revealed that the neural correlates of the perceptual illusion arose relatively late (EEG). In addition, a noticeable aspect of the 3T fMRI results is that BOLD signals showed stronger representation of the FGE in dorsal compared to ventral visual cortex (see in Supplementary Fig. 1). This might have resulted from asymmetric representation across the meridian of the visual field^32^.

Perception has long been considered an inferential process^3,33^, that retina inputs are modulated by spatiotemporal context and other priors to generate our perceptual experience. A number of neuroimaging studies have examined whether the neural signals in early visual cortex reflected the input properties or the perceived quality of the stimuli, with mixed results. Some studies showed that the BOLD signal in V1 reflected the perceived stimulus rather than the retinal input, such as activation reflecting distance scaling of perceived object size^34^ and activation along apparent motion trajectory where there was no direct stimulation^35^. Other studies have shown that local signals in V1 did not necessarily correspond to perceived brightness and color changes induced by modulating a surround field^36^. To reconcile the conflicting findings, an important point to consider is that BOLD responses are driven by both feedforward and feedback neural signals. In our study of FGE, the smaller estimated tilt angle based on fMRI signals in V1 could be due to a greater contribution from feedforward input signals in V1. In this regard, the layer-resolved 7T fMRI has a particular advantage, as shown in our results, in which the superficial layers tend to have more robust representations of the illusory tilt, compared to the middle layers that are more dominated by feedforward signals^22,23,25^.

Across individuals, EEG signal lateralization about 180 ms after flash onset closely correlated with the magnitude of FGE. But while all subjects showed illusory tilt effect in consistent directions, the corresponding (contra-ipsi) lateralized ERP was not always positive, with subjects experiencing weaker illusion tending to have little or reversed lateralization (Fig. 6b). This is likely because the observed ERP during that interval was also influenced by other sensory and cognitive processes. For example, a stronger feedforward representation may induce a larger negative component in the P1/N1 range, reducing the potential lateralized ERP signals in the time window. Another interesting observation is that the background by itself induced a significant lateralized EEG signal at around 120 ms (Fig. 4d), which was not observed when a vertically flashed bar was added to this background in the FGE condition. It is possible that the abruptly flashed bar attracted attention and reduced the signal from the rotating wedge background. Alternatively, it may have been canceled out by an oppositely lateralized signal from the perceived tilted bar, which means the illusory representation could have emerged as early as 120 ms after bar onset. The fact that the lateralized signal around 120 ms was not correlated with illusion size and did not outperform background-only condition in decoding implies that this signal was not intrinsically linked to the FGE. In any case, 120 ms is not typically considered in the temporal window of feedforward processing in early visual cortex. Overall, the temporal data strongly support a feedback interpretation of FGE.

An interesting observation is the below-chance level cross-decoding performance (from illusory to retinally tilted condition) shown in Fig. 5a. This was observed during a very early time window for the training stimulus. The implication is that the activity patterns of illusory (centered around 80 ms) and retinally tilted (centered around 100 ms) trials were likely oppositely lateralized. It is possible the two patterns represented different features of the stimuli. Indeed, the activity patterns of the illusory condition around the same time window cross decoded the background-only condition significantly above-chance, suggesting that the former was more related to the moving background wedge (note that the wedge would always be at the opposite side of the perceived location of the flashed bar, Fig. 4b). This below-chance decoding performance in the early time window of the illusory condition forms a clear contrast to the above-chance decoding in a later time window (~200 ms). Together, they point to an early background based and late illusory bar position based cross-decoding performance.

With the results from spatiotemporal imaging supporting a feedback interpretation of the FGE, the behavioral data showing that the perceived tilt in FGE could generate a TAE implies that the visual cortical neurons adapted to orientation representation driven by the feedback signals. Given that the goal of adaptation is to adjust the system’s sensitivity based on the statistics of the environment to process information more efficiently, this point becomes more interesting when the input driven feedforward representation and the feedback-driven perceptual representation are in conflict and both are available in cortex. When input signals and perceptual representation agree, it is difficult to distinguish between adaptation to feedforward or feedback signals. Our previous demonstration that orientation-selective adaptation could occur to invisible gratings^37,38^ constitutes support for adaptation to feedforward-dominated cortical representation of orientation. Our current results show that when the feedforward input orientation is different from perception, adaptation is primarily driven by the feedback-driven neural representation of the perceived property. These results also go beyond the demonstration of TAE from mentally generated bars^39,40^. Since no feedforward inputs were presented in those studies, there was no competition between the feedforward and feedback signals.

There were early experiments investigating the potential influence on adaptation effect resulting from dissociation between input and perceived properties of stimuli, with mixed results. For example, in the so-called flash-drag effect, where the perceived position of a flashed stimulus appears to be shifted in the direction of a nearby moving object, the perceived location biased the effectiveness of adaptation^41^. However, other studies showed that those motion-induced position changes had little contribution to the adaptation aftereffect^42,43^. The lack of clear results from these early studies could be due to weak adaptation effect^42^ or rather small size of perceptual mislocalization^43^. The FGE could induce a 10 times larger position shift compared with the flash-drag effect^7^, by presenting the flashed target on top of the moving background at the time it reverses its motion trajectory, rather than adjacent to the moving object. The current results, with complete dissociation between retinal input and perceived orientation of the adapting stimuli, combined with the clear demonstration of the feedback origin of the perceptual effect, provide unequivocal evidence for neural adaptation to feedback representations.

Since information processing networks consist of both hierarchical stages and parallel pathways, naturally adaptation could occur at multiple stages of processing. Consequences of adaptation observed at later stages of processing could be based on inherited signals from other parts of the neural networks, or the adaptation effect could be itself inherited^44^. For example, contrast adaptation effect could be observed in MT neurons or from the inheritance of contrast adaptation effect at early stages of processing^45,46^. Early studies have also demonstrated adaptation effect to biases in appearance in color and motion, which allowed the authors to conclude that these adaptation effects were cortical in origin^47–49^. In addition, attention could modulate the representational strength of attended features and in turn enhance its adaptation. While it is common that many factors modify the retinal input to generate perception, and these results are certainly consistent with adaptation to perception-linked neural representations, our current study has the advantage of explicitly contrasting the feedforward representation and feedback representation in their effectiveness for adaptation. Specifically, our study adds to the understanding of adaptation that when input signal and feedback representation are clearly different, the visual system can adjust its sensitivity based on the feedback-driven neural representation despite the discrepant feedforward representation. Although this point is demonstrated with just one perceptual phenomenon here, our study prompts future neural adaptation models to take into account the different roles of feedforward and feedback signals, especially when they are discrepant.

In summary, our spatiotemporal imaging results reveal that the illusory orientation representation was temporally late and spatially biased to the superficial cortical layers, thus pointing to a feedback origin of the FGE. Combined with psychophysical results, this study provides evidence that when perceived and input stimulus orientations of the adapting bars are dissociated with each other, the orientation adaptation mainly depends on the feedback supported neural representation linked to perception. These results highlight the important contribution of feedback signals for cortical neurons to recalibrate their sensitivity.

## Methods

### Participants

Eight healthy subjects (5 female, ages 21–27) participated in the psychophysics experiments; eleven (2 female, ages 21–27) participated in the 3T fMRI experiment (two subject was excluded due to head movment or failed to obtain clear retinotopy); seventeen (9 female, ages 22–35) participated in the 7T fMRI experiment; and twelve (4 female, ages 21–27) participated the EEG experiment (one subject was excluded due to excessive eye movement/blinks). Subjects were unaware of the purpose of the experiments. All observers had normal or corrected-to-normal vision and gave written consent. The protocol was approved by The Institutional Review Panel at the Institute of Biophysics (IBP), Chinese Academy of Sciences (CAS).

### Psychophysics stimuli and procedures

Subjects’ head position was stabilized with a chin rest at a viewing distance of 57 cm. Stimuli were presented in a dark room on a CRT monitor (NESO FS210A, Nanchang, China), with a resolution of 1024 × 768 and a refresh rate of 120 Hz. The experiment was programed in MATLAB (The MathWorks, Inc.) using the Psychophysics Toolbox^50,51^ extensions.

During the experiment, a small black fixation dot was presented at the center of the screen and a pair of rotating disks of 3.9 dva (degree of visual angle) radius were presented at the two sides of the fixation point, on a uniform gray background. The disks were patterned with 6-sectors (spanning 60° each sector). The distance between the fixation point and the center of each disk was 10.2 dva. The sectors had 25% Michelson Contrast^52^, which was defined by

1 $$C_{\rm{m}} = (L_{{\rm{max}}} - L_{{\rm{min}}})/(L_{{\rm{max}}} + L_{{\rm{min}}}),$$

where *L*_max_ and *L*_min_ and represent the luminance of brighter and darker sectors, respectively.

The disks rotated 250° (degrees of rotation) every second and reversed direction every 240 ms (covering 60°, 1 sector, in that time). On each reversal a light–dark edge would be at the vertical orientation, and for every other rotation reversal (480 ms/cycle) two red vertical bars (0.3 dva width) were flashed on for 33 ms, aligned with the light–dark edges.

In the first experiment, we tested the TAE to perceived tilted but retinally vertical condition. We first measured the size of the FGE. Subjects were presented with a pair of rotating sectored disks and two vertical bars were flashed briefly at the direction reversals. A pair of green pointers (0.3 dva) was presented around each of the two disks. Using the keyboard, the subjects adjusted the angles between the pointers until the pointers and bars appeared to be aligned. They had unlimited time to adjust the angles, and were asked to press the spacebar when they were satisfied with the angle alignment to record the setting and to start the next trial. The two rotation directions (left clockwise and right counter-clockwise, vice versa) were tested 5 times each for each subject. The mean perceived tilt (away from vertical) across subjects was 15.55° (*n* = 8, SD = 7.54).

The adaptation trial sequence is depicted in Fig. 1a. On each trial, subjects were presented with the same patterned disks as in the flash-grab measurement part of the experiment and adapted to the two flash bars. The bars were perceived to be tilted due to the FGE. The adaptation period included 11 flashes (5.3 s) in each trial, followed by a 33.3 ms blank period. Then a pair of test bars were presented for 33.3 ms. The test bars were the same as the pair of red bars presented during the adaptation period except that the angle between two bars was varied ranging from −6.9° to +6.9° (seven variations, −6.9°, −2.3°, −1.1°, 0°, +1.1°, +2.3°, +6.9°, positive degree represents the two bars converging upward). Subjects were asked to judge whether the two test bars were converging upward or downward using a 2AFC method. The seven different angular conditions of bars were tested 20 times each (selected in random order across trials).

Three control adaptation conditions were included in the experiment: (a) the vertical flashed bars only without the rotating background disks; (b) the rotating background disks only; (c) tilted flashed bars as in conventional TAE experiment (The bars were tilted 5.7° away from vertical). The tilted flash bars conditions and the flash-grab conditions are counterbalanced between blocks among the subjects.

In the seconed experiment, we tested the TAE to perceived vertical but retinally tilted condition. The conditions were similar to that described above, except that subjects needed to adjust the reversal angle of disks until the two flashed bars appeared vertical using keyboard. Subjects had unlimited time to make the adjustment. When they were satisfied with the adjustment, they pressed spacebar to start another trial. Two rotation directions were tested 20 times each for each subject. The mean orientation away from vertical across subjects was 16.02° (*n* = 8, SD = 7.34). The adaptation stimulus used in this experiment is demonstrated in Fig. 1b (right column).

The TAE was measured with similar procedure as described above, except that the adapting stimuli were retinally tilted but perceived vertical for each subject. Two control conditions were included as well, one is the vertical flashed bars without the background, and the other is the retinally tilted bars without the background as in conventional TAE experiments.

### 3T fMRI procedures and data acquisition

Stimuli were presented with an MRI safe projector (1024 × 768@60 Hz) on a translucent screen behind the head coil. For the FGE experiment, the rotating pinwheel background (Fig. 2a) was presented at 3.12% contrast, 36.87° of visual angle in diameter, rotating at 180° per second and changed motion direction every 0.67 s (120° per rotation). A red vertical bar (36.87 and 0.96° in length and width, respectively) was briefly presented for 67 ms at the boundary of two disc sectors, at the moment of background motion reversal. Subjects were instructed to keep fixation while passively viewed the stimuli. Four runs of functional data were collected for the FGE experiment, each consisted of 144 image volumes. Retinotopic localizer were rotating wedge and expanding ring checkerboard stimuli reversing contrast at 5 Hz. The wedge stimulus has a center angle of 22.5°, rotating clockwise across the full visual field in 32 s. The ring stimulus expanded from fixation to the edge of the viewing aperture (47.93° in diameter) in 32 s. Two runs of functional images were collected for the retinotopic localizer, 128 image volumes for each run.

MRI data were acquired with a 3T MRI scanner (Siemens Trio) using a 12-channel receive head coil at Beijing MRI Center for Brain Research (BMCBR), IBP, CAS. Functional images were acquired with a gradient echo planar imaging sequence (3 mm isotropic voxels, 30 axial slices of 3 mm thickness, 64×64 matrix with 3 mm in-plane resolution, TR/TE = 2000/28 ms, flip angle = 90°). High-resolution anatomical volume was obtained with a T1-MPRAGE sequence (1 mm isotropic voxels, 192 sagittal slices of 1 mm thickness, 256 × 256 matrix with 1 mm in-plane resolution, TR/TE = 2600/3.02 ms, flip angle = 8°).

### 7T fMRI procedures and data acquisition

Viewing aperture of the 7T screen was 26.27° horizontally and 19.85° vertically. Fullfield rotating pinwheel background (Supplementary Fig. 2) was presented at 2.91% contrast, rotating at 240° per second and changed motion direction every 0.5 s (120° per rotation). A red horizontal bar (26.27° and 0.52° visual angle in length and width, respectively) was briefly presented for 67 ms at the boundary of two disc sectors, at the moment of reversal of background motion. Subjects were instructed to keep fixation while passively viewed the stimuli. Nine runs of functional images were collected for the FGE experiment, 144 volumes of images for each run. Retinotopic localizer was a rotating bar stimulus with checkerboard patterns reversing contrast at 5 Hz (26.27° and 0.52° visual angle in length and width, respectively). Centered on the fixation, the bar rotated counter-clockwise from −16° to +15° in 32 s. Three runs of functional images were collected for the retinotopic localizer, each consisted of 128 volumes of images.

MRI data were acquired with a 7T whole body MRI scanner (Siemens Healthineers GmbH, Erlangen, Germany) using a 32 channels head coil (Nova Medical, Wilmington, USA) at BMCBR, IBP, CAS. For the first seven subjects, a reduced-FOV Gradient-echo EPI sequence was used to acquire functional images (0.85 mm isotropic voxels, 21 coronal slices of 0.85 mm thickness, 126 × 96 matrix with 0.85 mm in-plane resolution, TR/TE = 2000/21 ms, flip angle = 80°, 6/8 phase partial Fourier (GRAPPA acceleration factor 3). High-resolution anatomical volume was obtained with a T1-weighted MPRAGE sequence (0.7 mm isotropic voxels, 256 sagittal slices at 0.7 mm thickness, 320 × 320 matrix with 0.7 mm in-plane resolution, TR/TE = 3100/3.56 ms, TI = 1200 ms, flip angle = 5°) and a proton density or PD-weighted MPRAGE sequence (0.7 mm isotropic voxels, 256 sagittal slices at 0.7 mm thickness, 320 × 320 matrix with 0.7 mm in-plane resolution, TR/TE = 2340/3.56 ms, flip angle = 5°). For the rest ten subjects, functional images were collected with a GE-EPI sequence with larger FOV (TR = 2000 ms, TE = 23 ms, 80° flip angle, voxel size 0.8 × 0.8 × 0.8 mm, FOV 128 × 128 mm, 31 oblique-coronal slices, 6/8 phase partial Fourier, GRAPPA acceleration factor 3). High-resolution anatomic volume was obtained with a T1-weighted MP2RAGE sequence (TR = 4000 ms, TE = 3.05 ms, voxel size 0.7 × 0.7 × 0.7 mm, field of view 224 × 224 mm, 256 sagittal slices, receiver bandwidth 240 Hz/pix, 7/8 phase partial Fourier, 7/8 slice partial Fourier, TI1 = 750 ms, 4° flip angle, TI2 = 2500 ms, 5° flip angle).

### EEG procedures and data acquisition

Observers were tested individually in a dark testing room. Head position was stabilized with a chin rest at a viewing distance of 57 cm. Stimuli were presented on a CRT monitor (NESO FS210A, Nanchang, China) with a resolution of 800*600 and a refresh rate of 100 Hz. The experiment script was written in MATLAB (The MathWorks, Inc.) using the Psychophysics Toolbox^50,51^ extensions.

As shown in Fig. 4a, b, the screen was filled with a uniform gray background. A small, black fixation dot was 5.9 dva (degrees of visual angle) above the screen center and a 60° sector (6.3% contrast with background) of 15.6 dva radius rotated back and forth below the fixation point. The sector rotated 80° (degrees of rotation) every second and reversed direction every 1500 ms (covering 120°, from −60° to 60° around vertical meridian). When the reversal occurred, a green vertical bar (0.3 dva in width) might flash for 30 ms (3 frames) at the vertical meridian, aligning with one of the two edges of the sector.

In order to match the illusorily and retinally tilted conditions, we first did a psychophysical experiment to measure the size of FGE. Within each trial, the flashed bar was always illusorily titled toward one direction. The oscillating sector described above could be rotated clockwise or counter-clockwise using keyboard by the subjects, who were instructed to adjust the display so that the flashed bar appeared to be subjectively vertical. They had unlimited time to make this “subjective vertical” adjustment. When they were satisfied with the adjustment, they pressed spacebar to move on to the next trial. The two reversal directions were tested 20 times each for each subject.

In the EEG experiment, subjects were presented with the same rotating sector as in the psychophysics session, except that the bar always flashed at the vertical meridian (See Fig. 4). The green vertical bar had 50% chance to flash on for 30 ms at the reversal. The FGE biased the perceived location of the flash bar in the direction of the sector’s motion after the reversal. There were four situations after a reversal: (1) sector rotated to the left without bar flash; (2) sector rotated to the right without bar flash; (3) sector rotated to the left with the flashed bar perceived to be tilted to the left; (4) sector rotated to the right with the flashed bar perceived to be tilted to the right. (1) and (2) were termed “background-only” condition, whereas (3) and (4) were termed “illusory” condition. Stimuli were presented in runs that lasted ~120 s. Data from 5 runs were collected, yielding 200 repetitions in each situation. In the control experiment, only the retinally tilted flash bar was presented (adopting the angle obtained in the psychophysics session, 50% chance to flash), without the rotating background sector, termed “retinally tilted” condition.

EEG data were acquired from 64 scalp electrodes (Neuroscan), digitized at 1000 Hz. Vertical electro-oculogram (VEO) was recorded by electrodes placed above and below the left eye. Horizontal electro-oculogram (HEO) was recorded by electrodes placed at the left and right outer canthi. The reference electrode was placed on the top of the midline between electrodes CZ and CPZ.

### Psychophysics data analysis

Psychophysical data were analyzed using custom MATLAB scripts (MathWorks Inc.). The average behavioral performance was plotted separately for each condition as the percentage of upward responses against intersection angles of test bars (Fig. 1c, e). Data points were fitted with the following logistic function to estimate the PSE (point of subject equality) where the test bars appeared parallel (both vertical).

2 $$p(x) = {\gamma} + \frac{{1 - \lambda - \gamma }}{{1 + {\rm{e}}^{ - \beta * (x - \alpha )}}},$$

where *x* is the intersection angle and *p*(x) is the percentage of upward response. α, β, λ, and γ are free parameters that were fitted using least squares estimation.

The magnitude of TAE was measured as half the distance of PSEs following adaptation in two opposite orientations.

### fMRI data analysis

3T MRI data were analyzed with Brain Voyager QX software package^53^ and Matlab (MathWorks Inc.). Functional images were motion corrected, low and high pass temporal filtered, and slice timing corrected. The high-resolution T1 volume was co-registered to the first volume of functional images, and transformed to Talairach space. General linear model was used to estimate fMRI responses to the flashed bars with clockwise and counter-clockwise illusions. The retinotopic mapping data was analyzed using a cross-correlation method embedded in BrainVoyager QX software package. 16 phase lags (every 2 s) was used to find the best fit of polar angle or eccentricity representation for each voxel. ROIs of early visual cortices (V1, V2, V3d/VP) were defined according to the retinotopic maps on inflated cortical surface. For each ROI, voxels were sorted and resampled into 360 bins according to their polar angle representations. Then the BOLD response of the flashed bar was plotted as a function of polar angle. From this response curve, the angular representation of a flashed bar was estimated separately for the upper and lower visual fields, defined as the polar angle that splits the area under the curve into two equal halves.

7T MRI data were analyzed with AFNI^54^, Freesurfer (Fischl, 2012), and custom Matlab/Python codes. Functional images were motion corrected and EPI distortion. The high-resolution T1 volume was co-registered to the mean volume of functional images. General linear model was used to estimate fMRI responses to the red bars with clockwise and counter-clockwise illusions. A cross-correlation method with 32 phase lags (every one second) was used to generate the polar angle retinotopic map of early visual areas V1/V2/V3. Pial and White Matter surfaces were reconstructed based on PD corrected T1 volume^55^. An equi-distance method was used to estimate the relative cortical depth of a voxel. The voxels in a ROI were sorted and resampled into three depth bins: superficial depth (0–0.4), middle depth (0.4–0.8), and deep cortical depth (0.8–1.0). The partition ratio was selected based on the thickness of cortical layers of human visual cortex^56^. Similar as the 3T data analysis, BOLD response to the flashed bar was plotted as a function of polar angel representation. To alleviate the draining veins effect of BOLD signal cross cortical layers, the min and max values of polar angle response curve was normalized to 0 and 1. The FGE illusory effect was calculated as the difference of normalized response between two illusory conditions (clockwise vs. counter-clockwise), averaged across two polar angle windows (voxels identified through independent localizer scan with preferred orientation tuning to −14° to −6° and 6° to 14°). The input representation index was calculated as the mean of normalized responses centered on the horizontal meridian (where voxels had preferred orientation tuning ranging from −4° to 4°). The polar angle windows were chosen to maximize the sensitivity of the index, because when pooling across all subjects/areas/layers, the difference between CW/CCW illusory conditions were most prominent around ±10° (i.e., for voxels with preferred orientation tuning around 10° or −10°). A small gap was left between these orientation windows to mitigate potential cross talk, and a slightly different gap did not qualitatively change the final results. The data with error bars are displayed as mean ± SEM. The *p* values < 0.05 were considered statistically significant. Within-subject confidence intervals were estimated according to the method described by Cousineau^57^.

### EEG data analysis

Data were analyzed using EEGLAB v13.3.2 (http://www.sccn.ucsd.edu/eeglab) and MNE v0.16.2 (https://martinos.org/mne/)^58^. Raw data were first filtered off-line with a 1-35 Hz bandpass filter. Data excursions exceeding 75 μV at electrode VEO (−100 to +300 ms) were excluded from analysis. Remaining epochs were separately averaged according to the stimulus conditions. To select electrodes for the C1 amplitude and latency analysis, grand averaged ERPs were made for each electrode and each condition but pooling all subjects. Five electrodes showing the largest C1 amplitudes were chosen for further analysis (posterior electrodes including P3, P5, PO5, PO7, O1). To quantify the C1 amplitude and latency for each stimulus and each subject, the waveforms at these five electrodes were first averaged to obtain a mean waveform.

Multivariate pattern analysis^59^ was conducted using scikit-learn 0.16.0 (http://scikit-learn.org/)^60^. Linear support vector machine classifiers were trained at each time point for each subject to predict to which side the flashed bar was retinally or perceived to be tilted, using preprocessed EEG data from all electrodes as features. For the background-only condition, we were predicting to which side a bar would be illusorily tilted if it was flashed as in the illusory condition, although the imaginary bar was not actually displayed. The decoding accuracy was estimated using a stratified 10-fold cross-validation procedure, and the regularization parameter C was set to 1.0. Each feature (electrode) was normalized to have zero mean and unitary standard deviation. To reduce the impact of random noise in single trials, we employed a mini-ERP approach. From all trials sharing the same label in the training set, k trials were randomly selected and averaged into a mini-ERP, which served as one training sample. The sampling process repeated until 1000 samples were generated and used to train the classifier. Similar procedure was used at test time except that the mini-ERP samples were derived from test set. We chose *k* = 9 in current analysis, leading to a 3-fold boost in SNR and hence more accurate and robust decoding.

Cross decoding was performed across different conditions and different time points. A separate SVM was trained using all trials in condition A at time *t*_A_, and tested using all trials in condition B at time *t*_B_. The average prediction accuracy of all subjects was recorded in a matrix at row *t*_A_ and column *t*_B_. To reduce computational burden, the EEG time series were decimated in time, and raw trial data instead of mini-ERP were used (i.e., *k* = 1) in this analysis.

The inter-subject correlation between either instantaneous or time-averaged ERP amplitude and TAE effect size was quantified with Pearson’s linear correlation coefficient. The lateralization potential evoked by the vertical bar was calculated by first subtracting ERP signals in ipsilateral electrodes from corresponding contralateral electrodes, and then contrasting illusory condition with background-only condition. The same set of posterior electrodes were selected as with the ERP analysis. The illusion size for each subject was obtained by pooling all measurements for both directions from the adjustment experiment for both directions. The mean ERP amplitude was averaged within the interval between 177 ms and 400 ms after bar onset for visualization purpose. The time interval was chosen according to the onset of significant instantaneous correlation and the interval of significant higher decoding accuracy in illusory condition compared with background-only condition.

The difference in time series were tested for statistical significance at population level using cluster-based permutation test^61,62^ which corrected for multiple comparisons. Values at individual time points were first subjected to mass univariate t-test with cluster-defining threshold set to *p* < 0.05 (or |*r* | > 0.5 for correlation analysis). The resulted contiguous suprathreshold intervals, in which statistics were of the same sign, were defined as clusters. For cross-decoding matrix, 2D clusters were defined on regular lattice. These clusters had to further pass a critical value in “cluster mass” before reported as significant. Cluster mass is the sum of *t* values in the cluster. The critical values were obtained with the following procedure: (1) randomly permute left or right labels for each subject, apply mass univariate *t*-test, calculate cluster mass for each cluster, and record the max and min cluster mass values; (2) repeat the above for 10,000 times or all possible permutations, and construct the empirical distribution for max and min values; (3) take the 97.5 and 2.5 percentiles of the max and min distributions, respectively, as the critical values for a two-tailed test. The confidence interval of population mean time courses as well as instantaneous inter-subject correlation was estimated using the bootstrap technique by resampling the subjects with replacement for 1000 times.

### Reporting summary

Further information on research design is available in the Nature Research Reporting Summary linked to this article.

## Supplementary information

Supplementary Information

Reporting Summary

## Data Availability

The data that support the findings of this study are available from the corresponding authors upon reasonable request. A reporting summary for this Article is available as a Supplementary Information file. Source data are provided with this paper.

## Source data

Source Data

## References

[1] Clifford, C. W., Rhodes, G. *Fitting the Mind to the World: Adaptation and After-effects in High-level vision* (Oxford University Press, 2005).

[2] Schwartz O, Hsu A, Dayan P (2007). Space and time in visual context. Nat. Rev. Neurosci..

[3] Pizlo Z (2001). Perception viewed as an inverse problem. Vis. Res..

[4] Albright TD, Stoner GR (2002). Contextual influences on visual processing. Annu. Rev. Neurosci..

[5] Gilbert CD, Li W (2013). Top-down influences on visual processing. Nat. Rev. Neurosci..

[6] Lamme VA, Roelfsema PR (2000). The distinct modes of vision offered by feedforward and recurrent processing. Trends Neurosci..

[7] Cavanagh P, Anstis S (2013). The flash grab effect. Vis. Res..

[8] Jin DZ, Dragoi V, Sur M, Seung HS (2005). The tilt aftereffect and adaptation-induced changes in orientation tuning in visual cortex. J. Neurophysiol..

[9] Gibson JJ, Radner M (1937). Adaptation, after-effect and contrast in the perception of tilted lines. I. Quantitative studies. J. Exp. Psychol..

[10] Fang F, Murray SO, Kersten DJ, He S (2005). Orientation-tuned fMRI adaptation in human visual cortex. J. Neurophysiol..

[11] Forte JD, Clifford CW (2005). Inter-ocular transfer of the tilt illusion shows that monocular orientation mechanisms are colour selective. Vis. Res..

[12] Suzuki, S., Clifford, C. & Rhodes, G. in *Fitting the Mind to the World.: Adaptation and After-Effects high-Level Vision* Vol. 2 (eds Clifford, C. & Rhodes, G.) 135–172 (Oxford University Press, Oxford, 2005).

[13] Liu T, Larsson J, Carrasco M (2007). Feature-based attention modulates orientation-selective responses in human visual cortex. Neuron.

[14] Thompson P, Burr D (2009). Visual aftereffects. Curr. Biol..

[15] Clifford CW (2014). The tilt illusion: phenomenology and functional implications. Vis. Res..

[16] Benucci A, Saleem AB, Carandini M (2013). Adaptation maintains population homeostasis in primary visual cortex. Nat. Neurosci..

[17] Blakemore C, Carpenter RH, Georgeson MA (1970). Lateral inhibition between orientation detectors in the human visual system. Nature.

[18] Clifford CW, Wenderoth P, Spehar B (2000). A functional angle on some after-effects in cortical vision. Proc. R. Soc. Lond. Ser. B: Biol. Sci..

[19] Kohler PJ, Cavanagh P, Tse PU (2017). Motion-induced position shifts activate early visual cortex. Front. Neurosci..

[20] Hogendoorn H, Verstraten FA, Cavanagh P (2015). Strikingly rapid neural basis of motion-induced position shifts revealed by high temporal-resolution EEG pattern classification. Vis. Res..

[21] Engel SA, Glover GH, Wandell BA (1997). Retinotopic organization in human visual cortex and the spatial precision of functional MRI. Cereb. Cortex (N. Y., NY: 1991).

[22] Kok P, Bains LJ, van Mourik T, Norris DG, de Lange FP (2016). Selective activation of the deep layers of the human primary visual cortex by top-down feedback. Curr. Biol..

[23] Muckli L (2015). Contextual feedback to superficial layers of V1. Curr. Biol..

[24] Klein BP, Fracasso A, van Dijk JA, Paffen CL, Te Pas SF, Dumoulin SO (2018). Cortical depth dependent population receptive field attraction by spatial attention in human V1. NeuroImage.

[25] De Martino F, Moerel M, Ugurbil K, Goebel R, Yacoub E, Formisano E (2015). Frequency preference and attention effects across cortical depths in the human primary auditory cortex. Proc. Natl Acad. Sci..

[26] Wagstyl K (2018). Mapping cortical laminar structure in the 3d bigbrain. Cereb. Cortex.

[27] Self MW, van Kerkoerle T, Goebel R, Roelfsema PR (2017). Benchmarking laminar fMRI: neuronal spiking and synaptic activity during top-down and bottom-up processing in the different layers of cortex. Neuroimage.

[28] Bastos AM, Usrey WM, Adams RA, Mangun GR, Fries P, Friston KJ (2012). Canonical microcircuits for predictive coding. Neuron.

[29] Self MW, van Kerkoerle T, Super H, Roelfsema PR (2013). Distinct roles of the cortical layers of area V1 in figure-ground segregation. Curr. Biol..

[30] Van Kerkoerle T, Self MW, Roelfsema PR (2017). Layer-specificity in the effects of attention and working memory on activity in primary visual cortex. Nat. Commun..

[31] Felleman D, Van Essen D (1991). Distributed hierarchical processing in the primate cerebral cortex. Cereb. Cortex (N. Y., NY: 1991).

[32] Liu T, Heeger DJ, Carrasco M (2006). Neural correlates of the visual vertical meridian asymmetry. J. Vis..

[33] Helmholtz H. v. in *Science and Culture: Popular and Philosophical Essays* (ed. Cahan, D.) 127–203 (The University of Chicago Press, 1868).

[34] Murray SO, Boyaci H, Kersten D (2006). The representation of perceived angular size in human primary visual cortex. Nat. Neurosci..

[35] Muckli L, Kohler A, Kriegeskorte N, Singer W (2005). Primary visual cortex activity along the apparent-motion trace reflects illusory perception. PLoS Biol..

[36] Cornelissen FW, Wade AR, Vladusich T, Dougherty RF, Wandell BA (2006). No functional magnetic resonance imaging evidence for brightness and color filling-in in early human visual cortex. J. Neurosci..

[37] He S, Cavanagh P, Intriligator J (1996). Attentional resolution and the locus of visual awareness. Nature.

[38] He S, MacLeod DI (2001). Orientation-selective adaptation and tilt after-effect from invisible patterns. Nature.

[39] Mohr HM, Linder NS, Linden DE, Kaiser J, Sireteanu R (2009). Orientation-specific adaptation to mentally generated lines in human visual cortex. Neuroimage.

[40] Mohr HM, Linder NS, Dennis H, Sireteanu R (2011). Orientation-specific aftereffects to mentally generated lines. Perception.

[41] Kosovicheva AA, Maus GW, Anstis S, Cavanagh P, Peter UT, Whitney D (2012). The motion-induced shift in the perceived location of a grating also shifts its aftereffect. J. Vis..

[42] Fukiage T, Murakami I (2010). The tilt aftereffect occurs independently of the flash-lag effect. Vis. Res..

[43] Fukiage T, Murakami I (2013). Adaptation to a spatial offset occurs independently of the flash-drag effect. J. Vis..

[44] Solomon SG, Kohn A (2014). Moving sensory adaptation beyond suppressive effects in single neurons. Curr. Biol..

[45] Kohn A, Movshon JA (2003). Neuronal adaptation to visual motion in area MT of the macaque. Neuron.

[46] Kohn A (2007). Visual adaptation: physiology, mechanisms, and functional benefits. J. Neurophysiol..

[47] Goddard E, Solomon S, Clifford C (2010). Adaptable mechanisms sensitive to surface color in human vision. J. Vis..

[48] Zaidi Q, Sachtler W (1991). Motion adaptation from surrounding stimuli. Perception.

[49] Krauskopf J, Zaidi Q (1986). Induced desensitization. Vis. Res..

[50] Brainard DH, Vision S (1997). The psychophysics toolbox. Spat. Vis..

[51] Pelli DG, Vision S (1997). The VideoToolbox software for visual psychophysics: Transforming numbers into movies. Spat. Vis..

[52] Michelson, A. A. *Studies in Optics* (Courier Corporation, 1995).

[53] Goebel R, Esposito F, Formisano E (2006). Analysis of functional image analysis contest (FIAC) data with brainvoyager QX: From single-subject to cortically aligned group general linear model analysis and self-organizing group independent component analysis. Hum. Brain Mapp..

[54] Cox RW (1996). AFNI: software for analysis and visualization of functional magnetic resonance neuroimages. Comput. Biomed. Res..

[55] Van de Moortele P-F, Auerbach EJ, Olman C, Yacoub E, Uğurbil K, Moeller S (2009). T1 weighted brain images at 7 Tesla unbiased for Proton Density, T2* contrast and RF coil receive B1 sensitivity with simultaneous vessel visualization. Neuroimage.

[56] de Sousa AA (2009). Comparative cytoarchitectural analyses of striate and extrastriate areas in hominoids. Cereb. Cortex.

[57] Cousineau D (2005). Confidence intervals in within-subject designs: a simpler solution to Loftus and Masson’s method. Tutor. Quant. Methods Psychol..

[58] Gramfort A (2013). MEG and EEG data analysis with MNE-Python. Front. Neurosci..

[59] Grootswagers T, Wardle SG, Carlson TA (2017). Decoding dynamic brain patterns from evoked responses: a tutorial on multivariate pattern analysis applied to time series neuroimaging data. J. Cogn. Neurosci..

[60] Pedregosa F (2011). Scikit-learn: machine learning in Python. J. Mach. Learn. Res..

[61] Nichols TE, Holmes AP (2002). Nonparametric permutation tests for functional neuroimaging: a primer with examples. Hum. brain Mapp..

[62] Maris E, Oostenveld R (2007). Nonparametric statistical testing of EEG-and MEG-data. J. Neurosci. Methods.

