# Supplementary material for: Adaptation to feedback representation of illusory orientation produced from flash grab effect
Source: Nat Commun. 2020 Aug 6;11:3925. doi: 10.1038/s41467-020-17786-1 (PMC7411047; doi:10.1038/s41467-020-17786-1)
Supplement: Supplementary file 1 — Supplementary Information [file 41467_2020_17786_MOESM1_ESM.pdf]

Supplementary Information

Adaptation to Feedback Representation of Illusory Orientation Produced from Flash Grab Effect

Yijun et al.

Supplemental Figures

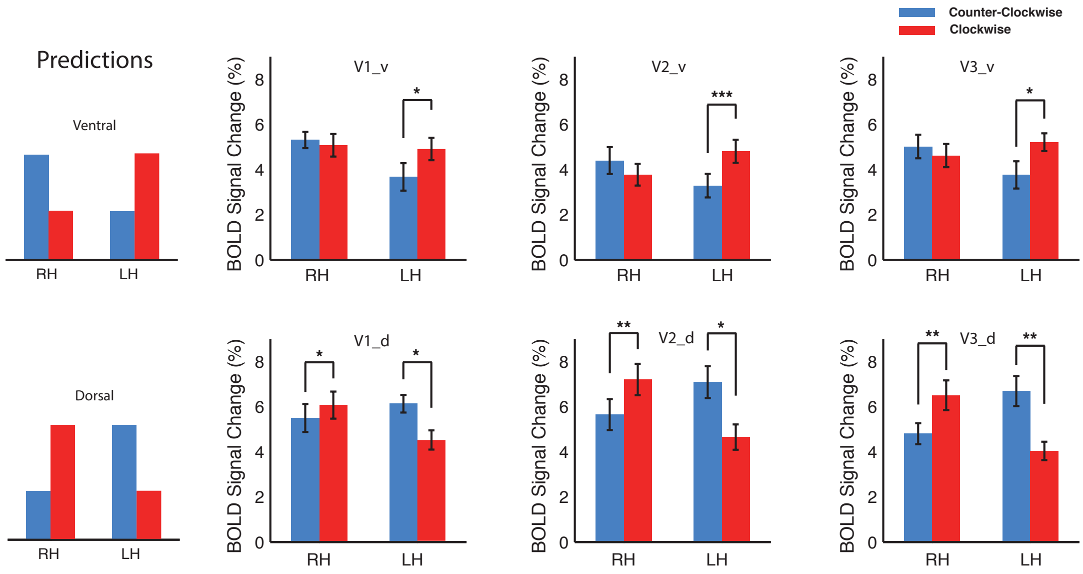

Supplementary Figure 1. Hemi-visual field fMRI response to the flash grab illusion. Upper row shows data from the upper visual field (ventral part of visual cortex); lower row shows data from the lower visual field (dorsal part of visual cortex). Compared to the counter-clockwise illusion, fMRI response to the clockwise tilted illusion was stronger in the left ventral and right dorsal visual cortex, but weaker in the left dorsal and right ventral visual cortex. The left column shows predictions for the illusory representation in the visual retinotopic cortex. The right three columns show the fMRI responses to the clockwise and counter-clockwise tilted illusion from different quadrants of the visual field in early visual cortices from V1 to V3. Three-way repeated measures ANOVA revealed a significant three-way interaction in V1 across dorsal/ventral, left/right hemisphere, and clockwise/counter-clockwise illusion (F (1, 8) = 38.11, $p < 0.001$). In the ventral part of V1 (corresponding to the upper visual field), compared to the counter-clockwise condition, clockwise illusion produced stronger fMRI signals in the left hemisphere (corresponding to the right visual field), and weaker response in the right hemisphere, resulting in a significant interaction between left/right hemisphere and clockwise/counter-clockwise illusion (F (1, 8) = 5.55, $p = 0.046$) in a two-way repeated measures ANOVA. The opposite was true for the dorsal part of V1: BOLD response to the clockwise condition was weaker in the left hemisphere and stronger in the right hemisphere (F (1, 8) = 14.16, $p = 0.006$). Similar results were found for V2 (V2_v/upper: F (1, 8) = 39.36, $p < 0.001$; V2_d/lower: F (1, 8) = 11.60, $p = 0.009$; three-way interaction: F (1, 8) = 39.83, $p < 0.001$), and V3 (V3_v/upper: F (1, 8) = 12.50, $p = 0.077$; V3_v/lower: F (1, 8) = 23.09, $p = 0.001$; three-way interaction: F (1, 8) = 28.56, $p < 0.001$). Simple effects of CW vs CCW conditions (stars in the figure, two-sided paired t-test) were not further corrected beyond the protection of a significant ANOVA. The error bars indicate standard error of mean (n=9 individuals). Source data are provided as a Source Data file.

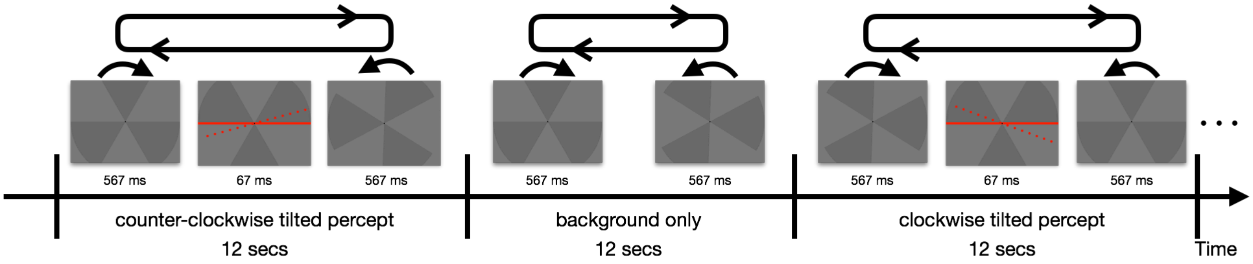

Supplementary Figure 2. Schematic diagram of stimuli and procedures for the 7T fMRI experiment**.** In each block, a red bar repeatedly presented(flashed) at the reversal point of the pinwheel disc which is rotating back and forth for 12 seconds as a constant background , alternating with 12 seconds rotating background-only stimulus section. The bar would be percieved as tilted clockwise or counter-clockwise from the horizontal meridian, depended on the direction of motion reversal. Red solid lines indicate the presented position of the bar, while red dotted lines illustrate the perceived position. The bar rotation covered a section of both left and right visual fields between 16 degrees clockwise and 15 degrees counter-clockwise from the horizontal meridian.

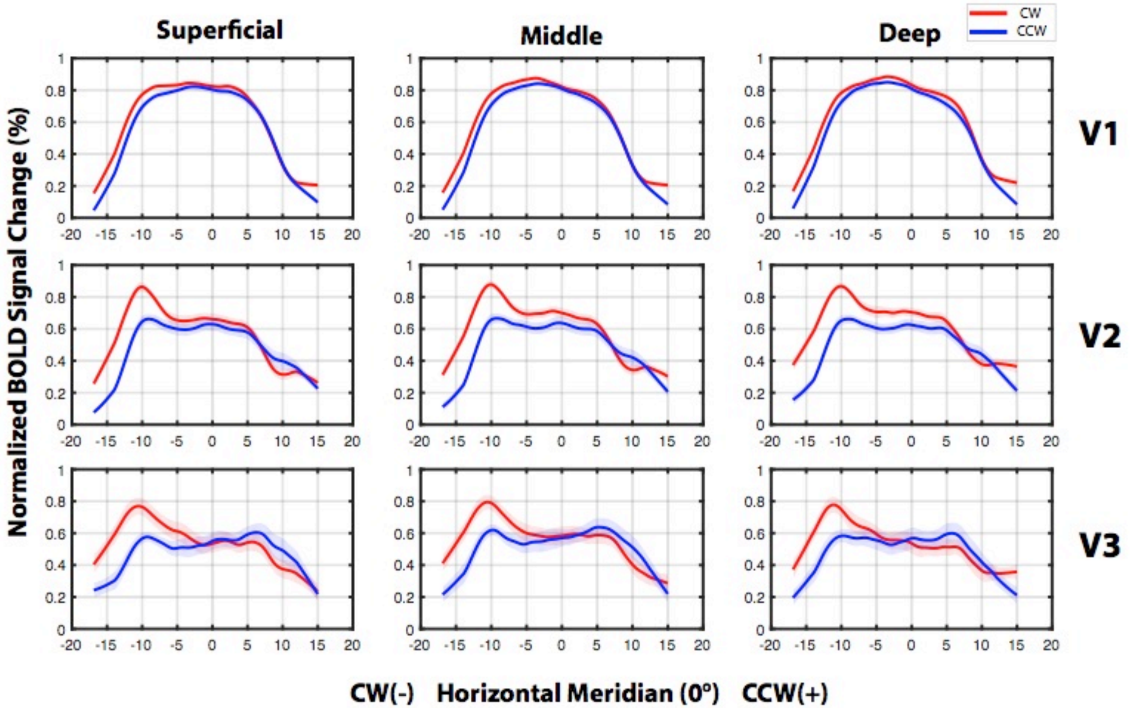

Supplementary Figure 3. Layer-specific bar angle representation of the flash grab illusion in each retinotopic visual area. In layers of V1, V2, V3, fMRI responses to the clockwise and counter-clockwise tilted illusions were plotted as a function of bar angle coordinates across the field of bar rotation. The red and blue curves represent mean retinotopic responses for clockwise and counter-clockwise conditions across seventeen subjects. The shading color indicate between-subject standard error. Source data are provided as a Source Data file.
