# Supplementary material for: Adaptation to feedback representation of illusory orientation produced from flash grab effect
Source: Nat Commun. 2020 Aug 6;11:3925. doi: 10.1038/s41467-020-17786-1 (PMC7411047; doi:10.1038/s41467-020-17786-1)
Supplement: Supplementary file 2 — Reporting Summary [file 41467_2020_17786_MOESM2_ESM.pdf]

nature research

Corresponding author(s): Sheng He, Lan Wang

Last updated by author(s): 7/2/2020

Reporting Summary

Nature Research wishes to improve the reproducibility of the work that we publish. This form provides structure for consistency and transparency in reporting. For further information on Nature Research policies, see Authors & Referees and the Editorial Policy Checklist.

Statistics

For all statistical analyses, confirm that the following items are present in the figure legend, table legend, main text, or Methods section.

n/a	Confirmed	
☐	☒	The exact sample size (*n*) for each experimental group/condition, given as a discrete number and unit of measurement
☐	☒	A statement on whether measurements were taken from distinct samples or whether the same sample was measured repeatedly
☐	☒	The statistical test(s) used AND whether they are one- or two-sided<br>*Only common tests should be described solely by name; describe more complex techniques in the Methods section.*
☒	☐	A description of all covariates tested
☐	☒	A description of any assumptions or corrections, such as tests of normality and adjustment for multiple comparisons
☐	☒	A full description of the statistical parameters including central tendency (e.g. means) or other basic estimates (e.g. regression coefficient) AND variation (e.g. standard deviation) or associated estimates of uncertainty (e.g. confidence intervals)
☐	☒	For null hypothesis testing, the test statistic (e.g. *F*, *t*, *r*) with confidence intervals, effect sizes, degrees of freedom and *P* value noted<br>*Give P values as exact values whenever suitable.*
☒	☐	For Bayesian analysis, information on the choice of priors and Markov chain Monte Carlo settings
☒	☐	For hierarchical and complex designs, identification of the appropriate level for tests and full reporting of outcomes
☐	☒	Estimates of effect sizes (e.g. Cohen's *d*, Pearson's *r*), indicating how they were calculated

Our web collection on statistics for biologists contains articles on many of the points above.

Software and code

Policy information about availability of computer code

Data collection	The experiment was programmed in MATLAB v2014b (The Math Works, Inc.) using the Psychophysics Toolbox v3.0.12.
Data analysis	fMRI data were analyzed using Brainvoyager QX 2.8 (http://www.brainvoyager.com), AFNI v18.0.24 (http://afni.nimh.nih.gov/), and Freesurfer v6.0.0 (http://freesurfer.net/) software packages, as well as calculations with Matlab v2014b (The Math Works, Inc.). EEG data were analyzed using EEGLAB v13.3.2 (http://www.sccn.ucsd.edu/eeglab), MNE v0.16.2 (https://martinos.org/mne/), and scikit-learn v0.16.0 (http://scikit-learn.org/).

For manuscripts utilizing custom algorithms or software that are central to the research but not yet described in published literature, software must be made available to editors/reviewers. We strongly encourage code deposition in a community repository (e.g. GitHub). See the Nature Research guidelines for submitting code & software for further information.

Data

Policy information about availability of data

All manuscripts must include a data availability statement. This statement should provide the following information, where applicable:

- Accession codes, unique identifiers, or web links for publicly available datasets
- A list of figures that have associated raw data
- A description of any restrictions on data availability

The source data underlying Figs. 1, 2, 3, 4, 5, 6 and Supplementary Figs. 1 and 3 are provided as a Source Data file. Source data are provided with this paper.

Field-specific reporting

Please select the one below that is the best fit for your research. If you are not sure, read the appropriate sections before making your selection.

☒ Life sciences ☐ Behavioural & social sciences ☐ Ecological, evolutionary & environmental sciences

For a reference copy of the document with all sections, see nature.com/documents/nr-reporting-summary-flat.pdf

Life sciences study design

All studies must disclose on these points even when the disclosure is negative.

Sample size	Sample sizes were selected based on similar previously published work and be adequate based on magnitude of effect. Comparable published studies of the flash grab effect, adaptation, and layer-resolved fMRI:<br>Cavanagh, P. & Anstis, S. The flash grab effect. Vision research 91, 8-20, doi:10.1016/j.visres.2013.07.007 (2013).<br>Kohler, P. J., Cavanagh, P. & Tse, P. U. Motion-induced position shifts activate early visual cortex. Frontiers in neuroscience 11, 168, doi:10.3389/fnins.2017.00168 (2017).<br>Fukiage, T. & Murakami, I. Adaptation to a spatial offset occurs independently of the flash-drag effect. Journal of vision 13, 7-7, doi:10.1167/13.2.7 (2013)<br>Kok, P., Bains, L. J., van Mourik, T., Norris, D. G. & de Lange, F. P. Selective activation of the deep layers of the human primary visual cortex by top-down feedback. Current Biology 26, 371-376, doi:10.1016/j.cub.2015.12.038 (2016).<br>Muckli, L. et al. Contextual feedback to superficial layers of V1. Current Biology 25, 2690-2695, doi:10.1016/j.cub.2015.08.057 (2015).
Data exclusions	One subject was excluded in the EEG experiment due to excessive eye movements/blinks in more than 50% of trials; Two subjects in 3T experiment were excluded due to massive head movement (fMRI data exclusion criteria: head movement > 3mm or showing unclear retinotopy).
Replication	No attempts were made to explicitly replicate data across different sample sets. Each experiment was replicated on each individual subject of reported subject group, with the effects replicated across subjects. Statistic analysis determined whether these effects were consistent across subjects.
Randomization	We used a within-subject design where all subjects were tested on same experimental conditions of each experiment. So, randomizing participants to different conditions was not necessary.
Blinding	All participants of each experiment were exposed to the same experimental conditions and unknown the purpose of the experiments. Thus blinding was not necessary.

Reporting for specific materials, systems and methods

We require information from authors about some types of materials, experimental systems and methods used in many studies. Here, indicate whether each material, system or method listed is relevant to your study. If you are not sure if a list item applies to your research, read the appropriate section before selecting a response.

Materials & experimental systems

n/a	Involved in the study	
☒	☐	Antibodies
☒	☐	Eukaryotic cell lines
☒	☐	Palaeontology
☒	☐	Animals and other organisms
☐	☒	Human research participants
☒	☐	Clinical data

Methods

n/a	Involved in the study	
☒	☐	ChIP-seq
☒	☐	Flow cytometry
☐	☒	MRI-based neuroimaging

Human research participants

Policy information about studies involving human research participants

Population characteristics	Eight healthy subjects (5 female, ages 21-27) unaware of the purpose of the experiment participated in the psychophysics experiments; eleven healthy adults (2 female, ages 21-27) participated 3T fMRI experiment (two subjects were excluded due to head movement or unclear retinotopy; another seventeen volunteers (9 female, ages 22-35) participated in the 7T fMRI experiment; and twelve subjects (4 female, ages 21-27) unaware of the purpose of the experiment participated the EEG experiment (one subject was excluded due to excessive eye movement/blinks). All observers had normal or corrected-to-normal vision and gave written consent.
Recruitment	Participants were randomly recruited from university undergraduates and graduates in Beijing, by advertising through social medias, such as BBS, Wechat, etc. All participants were screened to ensure that they had normal or corrected-to-normal vision,

	and were not on any psychotropic medication. The participants choose to participate based on a brief description of the experiment tasks. It is unlikely this caused a significant bias that may impact the results.
Ethics oversight	The protocol is approved by the Institutional Review Panel at the Institute of Biophysics (IBP), Chinese Academy of Sciences (CAS).

Note that full information on the approval of the study protocol must also be provided in the manuscript.

Magnetic resonance imaging

Experimental design

Design type	Block design
Design specifications	The 3T experiment consisted of 48 blocks per subject. The 7T experiment consisted of 108 blocks per subject. Each block was 12 seconds and the interval between blocks was 12 seconds.
Behavioral performance measures	In both the 3T & 7T experiment, subjects were instructed to keep fixation while viewing the flash grab stimuli.

Acquisition

Imaging type(s)	Functional, structural
Field strength	3 Tesla and 7 Tesla
Sequence & imaging parameters	3T: A gradient echo planar imaging (EPI) sequence was used to acquire functional images (3 mm isotropic voxels, 30 axial slices of 3 mm thickness, 64×64 matrix with 3 mm in-plane resolution, TR/TE = 2000/28 ms, flop angle = 90°).<br>7T: A T2*-weighted Gradient-echo ZOOPPA EPI sequence (working in progress, Siemens Healthineers GmbH, Erlangen, Germany) was used to acquire functional images (0.85 mm isotropic voxels, 21 coronal slices of 0.85 mm thickness, 126×96 matrix with 0.85 mm in-plane resolution, TR/TE = 2000/21 ms, flip angle = 80°).<br>T2*-weighted 2D gradient-echo EPI sequence (TR = 2000 ms, TE = 23 ms, 80° flip angle, voxel size 0.8 × 0.8 × 0.8 mm, field of view 128 × 128 mm, 31 oblique-coronal slices).
Area of acquisition	3T: whole brain; 7T: partial occipital lobe area.

Diffusion MRI ☐ Used ☒ Not used

Preprocessing

Preprocessing software	3T MRI data were preprocessed with Brain Voyager QX software package. 7T MRI data were preprocessed with AFNI software package.
Normalization	3T MRI data were non-linearly transformed. 7T MRI data were not normalized.
Normalization template	3T MRI data: Original Talairach space.
Noise and artifact removal	6 parameters rigid motion correction was performed on each subject.
Volume censoring	For the 3T data, volumes were censored when head movement (tanslation or rotation) larger than 3 mm. For the 7T data, a volume is censored if the motion derivative values have a Euclidean Norm above 0.3mm.

Statistical modeling & inference

Model type and settings	Retinotopy was established using mass univariate approach with standard phase encoding method. Polar angle representation was derived by a naive spatial encoding method.
Effect(s) tested	The effect of interest was the difference of reconstructed polar angle (3T) or normalized BOLD response (7T) between CW tilted and CCW tilted conditions.

Specify type of analysis: ☐ Whole brain ☒ ROI-based ☐ Both

Anatomical location(s)	Anatomical locations were determined based on fMRI retinotopic mapping (addtional fMRI scans were specific for this purpose).
Statistic type for inference (See Eklund et al. 2016)	Our core measurement was essentially a summary of multivariate activity within ROI, and thus was not subject to voxel or cluster selection issues.
Correction	No correction was applied to the reported ROI-based results.

Models & analysis

n/a	Involved in the study
☒	☐ Functional and/or effective connectivity
☒	☐ Graph analysis
☒	☐ Multivariate modeling or predictive analysis

October 2018
